# Gradual and Remarkable Tumor Shrinkage Following Seven-Fraction Stereotactic Radiosurgery Alone With a Marginal Dose of 48.3 Gy for Large Lobar Possibly Intra-sulcal Brain Metastasis From Renal Cell Carcinoma

**DOI:** 10.7759/cureus.36346

**Published:** 2023-03-19

**Authors:** Kazuhiro Ohtakara, Sachiko Aoki, Manabu Tajima, Takato Ohno, Kojiro Suzuki

**Affiliations:** 1 Department of Radiation Oncology, Kainan Hospital Aichi Prefectural Welfare Federation of Agricultural Cooperatives, Yatomi, JPN; 2 Department of Radiology, Aichi Medical University, Nagakute, JPN; 3 Department of Palliative Care Medicine, Kainan Hospital Aichi Prefectural Welfare Federation of Agricultural Cooperatives, Yatomi, JPN; 4 Department of Neurosurgery, Kainan Hospital Aichi Prefectural Welfare Federation of Agricultural Cooperatives, Yatomi, JPN

**Keywords:** volumetric modulated arc-based radiosurgery, tyrosine kinase inhibitor, stereotactic radiosurgery, renal cell carcinoma, large tumor, immune ckeckpoint inhibitor, fractionation, coagulopathy, brain metastasis

## Abstract

Brain metastases (BMs) from renal cell carcinoma (RCC) have the tendency of slow and insufficient tumor shrinkage along with prolongation of massive peritumoral edema following stereotactic radiosurgery (SRS). Herein, we describe a case of large lobar RCC-BM, with possible intra-sulcal location, treated with 7-fraction (fr) SRS without subsequent anti-cancer medication, which resulted in gradual and remarkable tumor shrinkage with extrication from the mass effect.

A 59-year-old woman was incidentally diagnosed with bilateral RCC associated with multiple lung metastases and subsequently presented with symptomatic single BM of 32 mm in the maximum diameter (9.54 cm^3^) two months later while vacillating. A biopsy of the kidney showed clear cell carcinoma. The patient was deemed medically inoperable for BM due to unfit conditions, including severe deep venous thromboses and thrombocytopenia. Considering the tumor volume, irregular tumor configuration, non-superficial location, and mass effect, 98% of the gross tumor volume (GTV D_98%_) was covered by 48.3 Gy in 7 fr with 64% isodose. Dose distribution was optimized with volumetric modulated arcs with the affirmative allowance of very inhomogeneous GTV dose. Anti-cancer medication was limited to nivolumab plus ipilimumab followed by everolismus 12 days before and during SRS, respectively. Subsequently, the patient transitioned to palliative care due to a declining general condition. Although long-term administration of steroids was required, gradual and marked tumor shrinkage (1.25 cm^3^, 13.1% of the initial volume) and mitigation of the peritumoral edema was observed during six months after SRS. The main location of the initial BM was deemed as intra-sulcal in the intraparietal sulcus and originated in the cerebral cortex. The patient died nine months after SRS.

The gradual but remarkable tumor response obtained with 7-fr SRS alone, in this case, provides a basis to further optimize fractionated SRS dosage to enhance efficacy and safety for large and/or symptomatic RCC-BMs not amenable to immediate surgical removal, in combination with anti-cancer pharmacotherapy, if feasible, including tyrosine kinase inhibitors, which may enhance efficacy against BM and mitigate adverse effects relevant to high dose SRS.

## Introduction

Single-fraction (sf) stereotactic radiosurgery (SRS) is a well-acknowledged treatment option for managing brain metastases (BMs) from renal cell carcinoma (RCC) [1]. Compared to those from lung or breast cancers, RCC-BMs have the tendency of gradual and inadequate tumor response associated with prolonged mass effect and peritumoral edema following sfSRS [2,3]. The local tumor control (LTC) probability or arrest of progressive tumor enlargement following sfSRS is generally high, whereas early and remarkable tumor shrinkage or nearly complete remission following sfSRS is rare [3,4]. Although prescription doses of sfSRS substantially vary from 16 Gy to 25 Gy between institutions [2-5], sfSRS with a sufficient marginal dose of ≥25 Gy to the gross tumor volume (GTV) margin can provide better tumor response and alleviation of perilesional edema [4]. In general, 24 Gy in a single fraction (fr) offers a one-year LTC probability of 95% for BMs ≤20 mm in their entirety [6]. However, given the limited brain tolerance to sfSRS [7], the acceptable indication of sfSRS for RCC-BM is restricted for tumors with GTV ≤2 cm, in particular, ≤15 mm or 1 cm^3^ for 24-25 Gy [8,9]. In addition, sfSRS with a modest marginal dose of ≤20 Gy for RCC-BM >2 cm considerably not only compromises the tumor response but also increases the risk of adverse radiation effects (ARE), including symptomatic brain radionecrosis [2,7-12]. Therefore, surgical removal has been usually prioritized for managing large symptomatic RCC-BM to immediately alleviate the neurological symptoms attributed to the mass effect [4,11]. However, certain RCC-BM cases, which are not amenable to open surgery due to compelling medical conditions, are expected to be treated with multi-fraction SRS (mfSRS) as a significantly less invasive alternative to open surgery [11,12]. There have been limited reports pertaining to mfSRS for RCC-BMs, and the dose/fractionation schemes along with dose distribution remain substantially variable [13,14]. In particular, the optimal dose/fractionation for achieving excellent tumor response and mitigation of peritumoral edema remains uncertain, especially for large tumors ≥30 mm [11,12,14]. Furthermore, limitations of brain tolerance for ≤5-fr SRS are being recognized [7,8,10].

Here, we describe a medically inoperable case of symptomatic large lobar RCC-BM (9.54 cm^3^), which was treated with 7-fr SRS without subsequent anti-cancer medication, which resulted in gradual and remarkable tumor shrinkage with adequate extrication from the mass effect during six months post-treatment. The main location of the BM was deemed as intra-sulcal with exophytic growth from near the cortex. This report was part of the clinical study approved by the Clinical Research Review Board of Kainan Hospital Aichi Prefectural Welfare Federation of Agricultural Cooperatives (20220727-1).

## Case presentation

**Figure 1:**
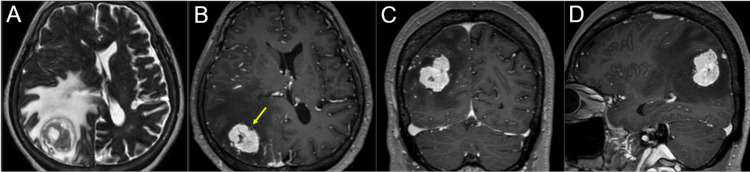
Magnetic resonance images before 7-fraction stereotactic radiosurgery. The images show T2-weighted image (WI) (A); contrast-enhanced (CE) T1-WI (B-D); axial views (A, B); coronal view (C); and sagittal view (D). (A-D) All images were co-registered on dedicated software and are shown in the same magnification and coordinates. A solid and irregular-shaped mass lesion (arrow in B) in the right parietal lobe is associated with massive peritumoral edema and mass effect.

A 59-year-old woman with a history of hypertension and diabetes was incidentally diagnosed with bilateral RCCs with diameters of 90 and 25 mm and associated with multiple lung metastases. Biopsy from the right kidney proved clear cell carcinoma with grade 1 nuclear atypia based on either Fuhrman’s criteria or the World Health Organization/International Society of Urological Pathology grading system. The hematological examination was unremarkable, except for secondary polycythemia and decreased prothrombin time [15], with intermediate risk according to the International Metastatic Database of Renal Carcinoma (IMDC) risk classification [1]. However, further evaluation followed by anti-cancer treatment was delayed due to patient-related compelling circumstances. The patient presented with left-sided hemiparesis two months after initial diagnosis, for which magnetic resonance imaging (MRI) determined a single BM (Figures 1A-1D).

The patient received first-line therapy with the immune checkpoint inhibitors (ICIs) ipilimumab and nivolumab the next day after BM diagnosis, along with proceeding with the preparation for surgical removal of the BM. However, the neurological and general condition deteriorated to the immobile state during 10 days after ICI administration. Further examination proved progressive thrombocytopenia (84,000/μL), elevated D-dimer level (73.1 μg/mL) and severe bilateral deep venous thromboses (DVT), which may have been triggered by the ICIs in the underlying high tumor burden-relevant coagulopathy [16]. Consequently, the patient was deemed medically inoperable at 10 days after initiation of ICIs, and direct oral anticoagulant (DOAC) was initiated. Following the referral regarding SRS, SRS was expeditiously initiated at 12 days after ICI treatment.

**Figure 2:**
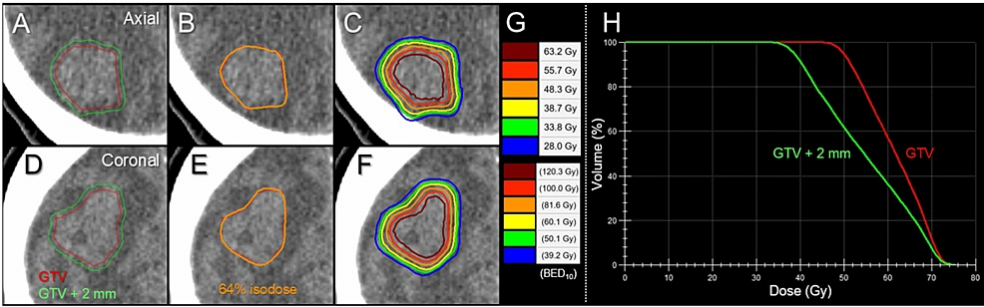
Target definition, dose distributions and dose-volume histograms for stereotactic radiosurgery. The images show axial views (A-C); coronal views (D-F); target definition (A, D); representative isodose distributions (B, C, E, F); representative isodose surfaces: absolute doses (upper) and the corresponding biological effective doses with an alpha/beta ratio of 10 (BED_10_) (G); dose-volume histograms (DVHs) (H). (A, D) A gross tumor volume (GTV) and an isotropic 2-mm margin-added object (GTV + 2 mm) for evaluation. (B, E) GTV and 64% (48.3 Gy) isodose surface (IDS) relative to the maximum dose. (C, F) The concentrically laminated steep dose gradients inside and outside the GTV boundary generated with volumetric-modulated arcs.

Considering the tumor volume, irregular configuration of the tumor boundary, non-superficial location [10,12], mass effect, and radiosensitivity for RCC, SRS ≤5 fr or ≥8 fr was deemed unsuitable for the BM, and 7-fr was adopted for the SRS [7,8,11,12]. The treatment device was a 5-mm leaf width multileaf collimator Agility® (Stockholm, Sweden: Elekta AB) mounted in a linac Infinity® (Stockholm, Sweden: Elekta AB) with a flattening filter-free mode of a 6 MV X-ray [17]. Dose distribution was optimized with volumetric modulated arcs (VMA) with the affirmative allowance of very inhomogeneous GTV dose to minimize the surrounding brain dose and to achieve better tumor response [11,12,17]. The arc arrangement consists of one coplanar arc and two non-coplanar arcs, which are allocated to divide the cranial hemisphere evenly [17]. The collimator angles for each arc are separately set to be 45, 90, and 135º. The planning system Monaco® (Stockholm, Sweden: Elekta) was used to optimize the VMA plan [17], for which 98% of the GTV (D_98%_) with 32 mm in the maximum diameter (9.54 cm^3^) was covered with 48.3 Gy in 7 fr, which was equivalent to a single dose of 24 Gy, based on the linear-quadratic (LQ) model-derived biological effective dose (BED) with an alpha/beta ratio of 10 (BED_10_) [11,12,17]. The GTV D_98%_ corresponded to 64% isodose surface (IDS) relative to the maximum dose. The dose distribution and relevant dosimetric parameters are shown in Figures 2A-2H and tabulated in Tables 1, 2.

**Table 1:** Dosimetric parameters of the gross tumor volume and relevant structures. D_X%_: the dose encompassing X% of the target volume; EQD2(2): equivalent total dose delivered at 2 Gy per fraction, based on the linear-quadratic model-derived biological effective dose with an alpha/beta ratio of 2 (BED_2_); GTV: gross tumor volume; D_max_: maximum dose

Evaluation index	D_98%_	EQD2(2)
GTV D_max_ (D_0.001 cc_)	75.4 Gy	240.7 Gy
GTV D_2%_	72.6 Gy	224.5 Gy
GTV D_50%_	61.8 Gy	167.3 Gy
GTV – 2 mm D_98%_	58.7 Gy	152.4 Gy
GTV – 1 mm D_98%_	53.4 Gy	128.5 Gy
GTV D_95%_	49.8 Gy	113.5 Gy
GTV D_98%_	48.3 Gy	107.5 Gy
GTV + 1 mm D_98%_	42.2 Gy	84.7 Gy
GTV + 2 mm D_98%_	36.9 Gy	67.1 Gy
GTV + 3 mm D_98%_	31.2 Gy	50.4 Gy

**Table 2:** Irradiated isodose volumes including the gross tumor volume. The IIVs of 100% and 50% of the GTV D_98%_ are 10.02 and 27.04 cm^3^, respectively. EQD2(2): equivalent total dose delivered at 2 Gy per fraction, based on the linear-quadratic model-derived biological effective dose with an alpha/beta ratio of 2 (BED_2_); IIV: irradiated isodose volume receiving a specific dose

Dose	EQD2(2)	IIV
46.4 Gy	100 Gy	10.88 cm^3^
43.7 Gy	90 Gy	12.00 cm^3^
40.8 Gy	80 Gy	13.47 cm^3^
37.8 Gy	70 Gy	15.01 cm^3^
34.6 Gy	60 Gy	17.03 cm^3^
33.2 Gy	56 Gy	17.98 cm^3^
28.0 Gy	42 Gy	22.37 cm^3^

The alpha/beta ratio of RCC for anti-tumor effect can be <10. Therefore, the BEDs and corresponding absolute doses in 7 fr and 1 fr equivalent to 24 Gy in 1 fr and 48.3 Gy in 7 fr, respectively, as a function of alpha/beta ratio, are tabulated in Table 3.

**Table 3:** Biological effective doses and corresponding absolute doses in 7 and 1 fraction(s) (fr) equivalent to 24 Gy in 1 fr and 48.3 Gy in 7 fr, respectively, as a function of alpha/beta ratio. BED: biological effective dose based on linear-quadratic model; fr: fraction; GTV: gross tumor volume; BED_2_: BED with an alpha/beta ratio of 2

Alpha/beta ratio	10	8	6	4	2
BED for 24 Gy in 1 fr	81.6 Gy	96.0 Gy	120.0 Gy	168.0 Gy	312.0 Gy
Equivalent absolute dose in 7 fr	48.3 Gy	50.5 Gy	53.1 Gy	56.0 Gy	59.5 Gy
GTV coverage	98.0%	93.4%	84.6%	73.0%	59.2%
BED_2_ (% to BED_2_ {312 Gy} for 24 Gy in 1 fr)	214.9 Gy (68.9%)	232.7 Gy (74.6%)	254.5 Gy (81.6%)	280.0 Gy (89.7%)	-
BED for 48.3 Gy in 7 fr	81.6 Gy	90.0 Gy	103.9 Gy	131.6 Gy	214.9 Gy
Equivalent absolute dose in 1 fr	24.0 Gy	23.1 Gy	22.1 Gy	21.0 Gy	19.8 Gy

**Figure 3:**
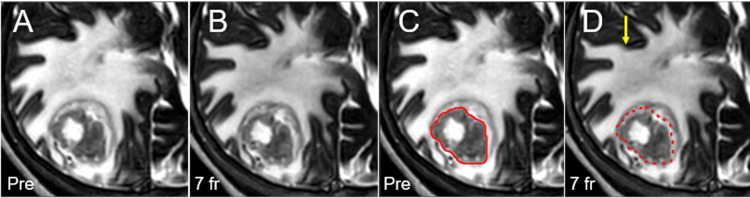
Magnetic resonance images obtained at one day before and at completion of stereotactic radiosurgery. Images show axial T2-WI (A-D); before SRS (A, C); at completion of SRS (7 fr) (B, D); the GTV contoured on the planning T2-WI (C); and the GTV contour (dashed) superimposed on the T2-WI at 7 fr. (A-D) All images were co-registered and are shown in the same magnification and coordinates. (B, D) At completion of 7 fr SRS, a slight dorsal displacement of the GTV without significant volume change is observed due to slight mitigation of the peritumoral edema (arrow in D). WI: weighted image; SRS: stereotactic radiosurgery; fr: fraction; GTV: gross tumor volume

Considering the risk of hemorrhage from RCC-BM under DOAC administration, everolimus as a second line therapy was initiated two days after initiation of SRS. T2-weighted images (T2-WI) were acquired at completion of SRS to evaluate potential tumor changes and/or deviation during SRS and showed no obvious change of the tumor configuration [12,18], although a slight dorsal displacement of the tumor owing to slight mitigation of the peritumoral edema was observed (Figures 3A-3D).

**Figure 4:**
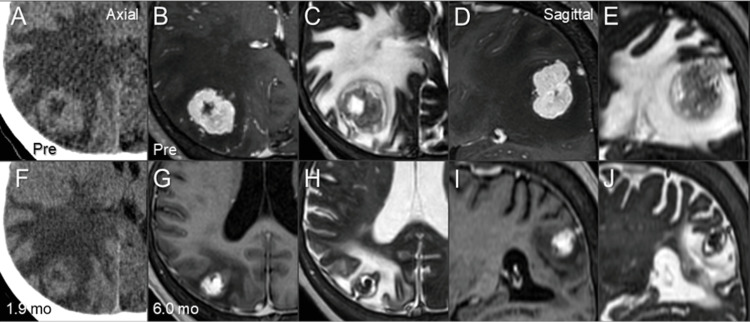
Computed tomography and magnetic resonance images before and after stereotactic radiosurgery. The images show axial images (A-C, F-H); sagittal images (D, E, I, J); computed tomography images (A, F); CE-T1-WI (B, D, G, I); T2-WI (C, E, H, J); before SRS (A-E); at 1.9 months after SRS (F); and at 6.0 months (G-J). (A-J) All images were co-registered and are shown in the same magnification (A-J) and coordinates (A-F). (F) At 1.9 months after SRS, considerable shrinkage of the BM was observed along with modest alleviation of the perilesional edema and mass effect. (G-J) At 6.0 months, marked shrinkage of the BM and mitigation of the peritumoral edema were observed along with extrication from the mass effect. CE: contrast-enhanced; WI: weighted image; SRS: stereotactic radiosurgery; BM: brain metastasis

Although thrombocytopenia and elevated D-dimer improved to 166,000/μL and 14.2 μg/mL, respectively, the neurological and general condition of the patient remained stable at SRS completion. Considering the prolonged decline in the medical condition of the patient, everolimus was discontinued the next day after SRS completion. The patient transitioned to palliative care due to persistence of severe hemiparesis and DVT. Although prolonged administration of steroids was required, gradual and remarkable shrinkage of the BM (1.25 cm^3^, 13.1% of the initial volume) with extrication from the mass effect was confirmed at 1.9 and 6.0 months (Figures 4A-4J).

**Figure 5:**
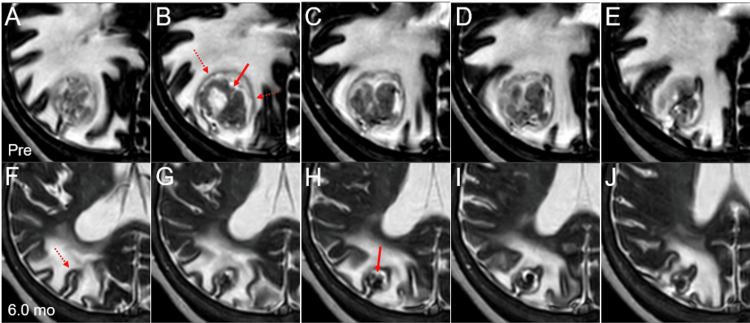
T2-weighted images before and six months after stereotactic radiosurgery. The images show axial T2-WIs (A-J); before SRS (A-E); and at 6.0 months after SRS (F-J). (A-J) All images were co-registered and are shown in the same magnification and coordinates. (A-E) An irregularly-shaped mass (arrow in B) is surrounded by a slightly high-intensity rim (dashed arrows in B), with a high-intensity structure, possibly trapped cerebrospinal fluid, intervening these structures. (F-J) At 6.0 months after SRS, the regressed low-intensity irregular mass with continuity to part of the cortexes is mainly located between the cortexes in the intraparietal sulcus (dashed arrow in F). WI: weighted image; SRS: stereotactic radiosurgery

Retrospective review of T2-weighted images before and after SRS along with other MRI information suggested that the main location of the BM was intra-sulcal, with exophytic growth from the origin at the cerebral cortex (Figures 5A-5J).

Neurological symptoms gradually improved, and the patient was discharged from the palliative care ward temporarily at 3.4 months after SRS. Five months after SRS, the patient gradually presented with sluggishness and motor dysphasia, which was attributed to a newly emerged BM in the left frontal lobe (data not shown). Therefore, 5-fr SRS was implemented for the BM with 32 mm in the maximum diameter (7.69 cm^3^) at 6.1 months, after which protracted administration of steroids was continuously required. Imaging evaluation thereafter was unavailable. The patient required oxygen at 7.7 months and died at 9.0 months after SRS, mainly due to extracranial disease progression.

## Discussion

For large symptomatic RCC-BM concomitant with prominent peritumoral edema and mass effect, we have prioritized surgical removal, if feasible, because it is superior to sfSRS or mfSRS in terms of immediate amelioration of the relevant symptoms attributed to the mass effect [4]. However, the development and establishment of an efficacious and less invasive alternative treatment for large RCC-BM not amenable to surgical removal is important [11,12]. During 20 years of the first author’s experience with SRS for BM, the present case was the most challenging RCC-BM. Since 2018, to improve anti-BM efficacy and safety irrespective of tumor volume, prescribed doses equivalent to BED_10_ ≥80 Gy have been preserved for GTV margins (D_≥98%_) with 3-15 fr according to tumor volume, location, and distance among multiple BMs [10-12,17]. Considering the limited brain tolerance to SRS ≤5 fr, ≥6 fr was deemed suitable for the present case [7,8,17]. Additionally, a high dose per fr may be advantageous for RCC, as the alpha/beta ratio of RCC can be <10. Although we have commonly used ≥8 fr for BM ≥30 mm [12,17], 7 fr was applied in the present case.

Among the previous reports regarding mfSRS for large RCC-BM [1,6,13,14], Sinclair et al. reported a noteworthy case of two large symptomatic RCC-BMs of 11.95 and 17.34 cm^3^ metachronously treated with 3-fr SRS over seven days with response-adaptive planning for each fraction using Leksell Gamma Knife (LGK) [14]. The marginal doses to GTVs were 8.5-9.5 Gy/fr with 35%-40% IDSs, which are extremely inhomogeneous even for LGK, with the GTV coverage of 10 Gy/fr being 84%-96%. Transient tumor expansion at 2 fr was observed for both BMs with 110.0% and 109.9% of the initial volume, respectively. Subsequent systemic therapy included sunitinib, nivolumab, and axitinib in sequence up to 14 months after the first SRS. The first GTV of 11.95 cm^3^ with 40% IDS covering showed sustained tumor expansion (105.8%) three months after SRS, with subsequent gradual response to 32.6% and 8.4% of initial tumor volume at six and 20 months, respectively. In addition, the second GTV of 17.34 cm^3^ with 35% IDS covering showed rather early response with 91.9% at 3 fr and 66.0%, 42.0%, and 18.5% at one, two, and eight months, respectively. The differences in tumor response may be attributed to those in % IDS for GTV covering, i.e., more inhomogeneous GTV dose for the latter, anti-cancer medication, and the dominancy of solid tumor component for the latter. However, the BED for 30 Gy in 3 fr is considerably low compared to that for 48.3 Gy in 7 fr, irrespective of the alpha/beta ratio. Sequential administration of tyrosine kinase inhibitors (TKIs) and ICI would contribute to enhanced tumor responses [5,19].

The present case showed no obvious tumor swelling or shrinkage during SRS [12,18]. Furthermore, despite discontinuation of anti-cancer medication after SRS, the BM of 9.54 cm^3^ showed a gradual and remarkable volume reduction to 13% with sufficient extrication from the pre-existing mass effect during six months. However, amelioration of relevant neurological symptoms was far from early and adequate palliation [11,12]. Therefore, long-term steroid administration was required. To further enhance efficacy, and especially earlier and more superior tumor response, dose escalation of the GTV marginal dose and combined administration of anti-cancer medications including TKI and/or ICI concurrently and/or sequentially were considered [5,19]. If the alpha/beta ratio of RCC is assumed to be ≤6-8, the single dose equivalent to 48.3 Gy in 7 fr is ≤22.1-23.1 Gy as shown in Table 3. Moreover, the absolute doses in 7 fr are equivalent to a single fr of 25 Gy for the alpha/beta ratio of 6-8 corresponds to 53-55.6 Gy [4]. The GTV D_98%_ ≥50.5-53.1 Gy in 7 fr with BED_6-8_ ≥96-120 Gy, equivalent to 24 Gy in 1 fr, may provide better tumor response in the present case, while the risk of ARE inevitably increases. TKI and ICI can provide efficacy for existing BMs, and therefore continued administration following SRS may enhance tumor response [1,5,19]. These regimens are also anticipated to enhance intra- and extra-cranial overall disease control for metastatic RCC [1,5,19]. Furthermore, vascular endothelial growth factor receptor (VEGFR) TKI can mitigate AREs relevant to high-dose SRS [20]. Early combined use of mfSRS and VEGFR-TKI may further alleviate the peritumoral edema and reduce steroid dependence.

The main location of the BM was initially considered as the deep white matter (location grade 2) [8,10,12], although it proved to be intra-sulcal with exophytic growth from the cerebral cortex. The substantive extra-axial location without the direct involvement of the white matter may not be susceptible to late ARE [10,12]. However, the superficial extra-axial location can involve nearby vessels, in which the involvement of the superficial vein may lead to venous congestion followed by the aggravation of the affected brain edema.

Inherent limitations in the present report include a lack of MRI evaluations between SRS completion and six months after SRS, the limited imaging follow-up period of six months for evaluating long-term efficacy and safety, and lack of intra-operative and pathological findings to verify the tumor condition at six months and its accurate location. Nevertheless, the present case forms the basis for further investigation to establish optimal mfSRS for RCC-BM deemed not amenable to sfSRS. SRS specialists should be committed to continuing to optimize mfSRS to consolidate efficacy and safety for large symptomatic RCC-BM.

## Conclusions

Large symptomatic lobar 32-mm BM from RCC was treated with 7-fr SRS with the GTV margin being covered with 48.3 Gy, BED_10 _81.6 Gy, with 64% IDS, which resulted in gradual and remarkable tumor shrinkage with sufficient extrication from the mass effect, despite discontinuation of anti-cancer medication after SRS. The main location of the BM was deemed as intra-sulcal with exophytic growth from the cerebral cortex. The present case provides the basis for further optimization of mfSRS, with combined and synergistic utilization of anti-cancer pharmacotherapy, including TKI and ICI.
